# Implementing an Internal CLABSI Validation Program

**DOI:** 10.1017/ash.2024.128

**Published:** 2024-09-16

**Authors:** Lauren DiBiase, Lisa Teal, Emily Sickbert-Bennett Vavalle, Lisa Stancill, Cyndi Culbreth, Karen Croyle, Natalie Schnell, Katherine Schultz, Tara Sotak, Jessica Wiley, Karen Graham

**Affiliations:** UNC Health; UNC Medical Center; UNC Health Care; UNC Hospitals

## Abstract

**Background:** The National Healthcare Safety Network (NHSN) provides detailed surveillance case definitions for healthcare-associated infections (HAI), including central line-associated bloodstream infections (CLABSI). CLABSI data are used for several purposes, including improving patient safety, value-based purchasing, and comparing hospitals’ performance. Our Infection Prevention (IP) team conducts house-wide HAI surveillance. To ensure that our hospital CLABSI reporting is accurate and that staff are implementing case definitions consistently and systematically, we conducted an internal validation of CLABSI. This undertaking allowed us to identify educational opportunities for IPs and improve surveillance data consistency. **Methods:** At UNC Hospitals, data on all positive blood cultures collected in the inpatient setting from July 2022 – June 2023 were obtained from electronic medical records. A random number generator was used to select 16 records per quarter. Each record was then randomly assigned to two different IPs (out of 8 total inpatient IPs) for review. Concordance of CLABSI classification was summarized across the two reviews and compared to the initial review. Discordant cases were then reviewed by the Associate IP Director (a certified IP with 15 years of experience) for final adjudication. A summary of findings and discordant cases details were discussed at regular IP educational meetings. **Results:** From July 2022-June 2023, there were 1658 positive blood cultures collected in the inpatient setting. Of the 64 randomly selected blood cultures, total concordance amongst all reviewers occurred 65.6% of the time. Concordance improved in the 2nd half of FY23 compared to the 1st half (72% vs, 59%, p>0.05). Amongst the 33% of blood culture results with reviewer discrepancy, the most common reasons were related to distinction of a bloodstream infection secondary to another infection site (32%) and application of the repeat infection timeframe (18%). Importantly, there was only one instance where a blood culture result was categorized by all 3 reviewers as present on admission, but upon Associate Director review, actually represented a CLABSI (i.e., false negative). **Conclusions:** Standardized case definitions remain open to interpretation. At our hospital, we experienced discordance in approximately one-third of instances during review of blood culture data amongst trained infection preventionists. Reviewing all blood culture data is key for validation so that both false positives and false negative CLABSIs can be identified. Identifying the most common reasons for discordance and using specific examples when case disagreement occurred for educational purposes may lead to improved reliability and accuracy of application of the NHSN surveillance defintions.

